# An investment case: the role of advocacy in addressing discrimination of vulnerable and marginalized populations at risk for HIV in sub‐Saharan Africa

**DOI:** 10.1002/jia2.25719

**Published:** 2021-06-30

**Authors:** Nerima Were, Felicita Hikuam, Ishtar Lakhani, Berry D Nibogora, Makhosazana Mkhatshwa

**Affiliations:** ^1^ Kenya Legal and Ethical Issues Network on HIV and AIDS (KELIN) and School of Law College of Humanities and Social Sciences at the University of Nairobi Nairobi Kenya; ^2^ AIDS and Rights Alliance for Southern Africa (ARASA) Windhoek Namibia; ^3^ Sex Worker Rights Activist Cape Town South Africa; ^4^ African Men for Sexual Health and Rights (AMSHeR) Johannesburg South Africa; ^5^ Treatment Action Campaign (TAC) Johannesburg South Africa

**Keywords:** advocacy, strategies, HIV, discrimination, marginalized, vulnerable

From the onset of the HIV epidemic, civil society advocacy has been central to addressing some of the most difficult barriers to improved HIV outcomes, including failures in service delivery, gender disparity and discrimination of vulnerable and marginalized populations at risk for HIV [1]. This Viewpoint discusses the role of advocacy in sub‐Saharan Africa, the region hardest hit by HIV, and highlights some examples showing how this work has contributed significantly to many important policy and structural reforms that have increased equitable access to HIV prevention, treatment, care and support services among all people, including the most vulnerable and marginalized. It concludes with a call for innovative new ways to fund such activities.

This advocacy has typically focused on increasing awareness, influencing public opinion, directing decision makers towards positive change and addressing social and structural barriers [2]. Some effective strategies used by civil society include community‐led monitoring; movement and coalition building; proposing and demanding specific legal, policy and practice reforms; and strategic litigation. Below are some examples of advocacy initiatives in the region that have proven successful.

In South Africa, the Treatment Action Campaign (TAC) was founded by people living with HIV (PLHIV) activists in 1998 to fight for access to HIV treatment, which at the time was not available in the public health system used by the majority of those in need. Among its notable early successes, in 2001, was a lawsuit against the health ministry that forced the government to expand access to nevirapine for HIV‐positive pregnant women to reduce HIV transmission risk to their infants [3]. This advocacy effort along with subsequent ones regarding ART access more broadly helped to save thousands if not millions of lives. More recently, one key component of TAC’s work is community‐led monitoring undertaken regularly by its over 8,000 members who volunteer [4], to collect data that is used to inform advocacy towards improved treatment and care for PLHIV. In a more recent example of the power of such advocacy, from 2014, TAC used information gathered about migrants being unable to receive services in health care facilities in Limpopo province to pressure stakeholders, including the Department of Home Affairs and South African Police Service [5]. These efforts resulted in the establishment of a Migrants’ Health Forum and migrants – many of whom living with or at high risk for HIV – being able to access health care in the public sector in the province [6].

Coordination and training are useful steps in overall advocacy processes towards greater impact, especially among marginalized populations that are stigmatized, criminalized and denied support and services. For example African Men for Sexual Health and Rights (AMSHeR), a regional coalition of men who have sex with men founded in 2009, focuses on building advocacy capacity across 18 member networks and groups. Between 2016 and 2020, AMSHeR organized convenings of gay men in eight countries to allow for socialization and information exchange, priority setting for national and local level advocacy and organizational development. Through this process, new leadership of young gay men emerged and has proceeded to represent their communities in influencing policy platforms that can deliver more health and human rights support for their communities. These include Global Fund to Fight HIV, Tuberculosis and Malaria country coordinating mechanisms and the US President’s Emergency Plan for AIDS Relief country operational plans [7].

Effective civil society advocacy in the region for the most marginalized and vulnerable is often highly specific and localized. In South Africa, one of the priorities of the Sex Workers Education and Advocacy Taskforce (SWEAT) since its founding in the early 2000s has been to decriminalize sex work, arguing that existing laws making all aspects of sex work illegal have increased their vulnerability to HIV and sexual violence [8]. Through a series of creative interventions, including a satirical sex worker‐led political party running in the national elections, SWEAT helped to ensure that decriminalization of sex work was included as a recommendation in the South African Government’s National Strategic Plan on Gender‐Based Violence and Femicide (March 2020) and that the governing political party acknowledged the need to decriminalize sex work in 2017 [9]. These are two important steps in ongoing efforts to decriminalize sex work.

These examples illustrate that achieving key civil society advocacy goals is often a lengthy, multi‐stage process that requires adequate human and financial resources at every step. Directly addressing financing constraints, one of the main barriers to scaling up and sustaining effective civil society advocacy in sub‐Saharan Africa, is critical for its potential impacts to be realized.

A strong investment case is needed to convince governments, donors and other stakeholders of the critical value of advocacy in addressing treatment and prevention gaps, human rights violations, gender disparity and discrimination of vulnerable and marginalized populations. Based on long‐term analysis of civic advocacy and its impacts in different contexts, this shows that investing in advocacy leads to longer‐term financial and programming efficiencies in HIV responses, including by reducing the need for costly health care for people denied access to treatment, unable to obtain necessary prevention services, or experiencing sexual and physical violence that is unchecked in part because of criminalization of victim’s behaviour [10].

The investment case may be founded on a dedicated funding mechanism for civil society advocacy that works to strengthen national and sub‐national advocacy [11]. Most funding, at least initially, would come from bilateral donors and foundations but this can be developed into a pooled funding mechanism that focuses on equitable distribution of funds to foster strong civic engagement in HIV and human rights. Local consortia of civil society could identify and outline priorities based on their most pressing needs. To reduce individual risk and provide cover for supporting work deemed highly sensitive, the pooled funding mechanism will allow engagement in a coordinated and collaborative manner to support these priorities, including the need for more core and operational funding [12].

These illustrations are just a handful of the many ways in which civil society advocacy has changed or laid the groundwork for reforms in policies, laws and practices that directly impact how and to what extent vulnerable and marginalized people can get the services and protection they need and deserve. In addition to making more financing available, with fewer strings attached, advocates in sub‐Saharan Africa also need more support from both inside and outside the region to force policymakers to act to remove existing barriers to advocacy that restrict their ability to operate freely and independently.

## Competing interests

The authors submit that there are no conflicting personal or financial relationships with any persons or organizations that have influenced the development of this paper. Each of the authors is representing both personal and organizational views on the subject matter and has the necessary approval to represent their organizations. The authors declare that we have no competing interest related to the development and publication of this commentary.

## Authors’ contributions

NW contributed to the conceptualization of the paper, framing of the investment case and was the overall drafter. FH contributed to the conceptualization of the paper and framing of advocacy and making an investment case. BDN developed the case study for men who have sex with men. IL developed the case study on sex workers. MM developed the section on movement building and community‐led monitoring.
